# The Transmissibility of Highly Pathogenic Avian Influenza in Commercial Poultry in Industrialised Countries

**DOI:** 10.1371/journal.pone.0000349

**Published:** 2007-04-04

**Authors:** Tini Garske, Paul Clarke, Azra C. Ghani

**Affiliations:** 1 Department of Epidemiology and Population Health, London School of Hygiene and Tropical Medicine, London, United Kingdom; 2 Institute for Mathematical Sciences, Imperial College London, London, United Kingdom; University of East Piedmont, Italy

## Abstract

**Background:**

With the increased occurrence of outbreaks of H5N1 worldwide there is concern that the virus could enter commercial poultry farms with severe economic consequences.

**Methodology/Principal Findings:**

We analyse data from four recent outbreaks of highly pathogenic avian influenza (HPAI) in commercial poultry to estimate the farm-to-farm reproductive number for HPAI. The reproductive number is a key measure of the transmissibility of HPAI at the farm level because it can be used to evaluate the effectiveness of the control measures. In these outbreaks the mean farm-to-farm reproductive number prior to controls ranged from 1.1 to 2.4, with the maximum farm-based reproductive number in the range 2.2 to 3.2. Enhanced bio-security, movement restrictions and prompt isolation of the infected farms in all four outbreaks substantially reduced the reproductive number, but it remained close to the threshold value 1 necessary to ensure the disease will be eradicated.

**Conclusions/Significance:**

Our results show that depending on the particular situation in which an outbreak of avian influenza occurs, current controls might not be enough to eradicate the disease, and therefore a close monitoring of the outbreak is required. The method we used for estimating the reproductive number is straightforward to implement and can be used in real-time. It therefore can be a useful tool to inform policy decisions.

## Introduction

A new highly pathogenic strain of avian influenza, H5N1, emerged in the poultry markets of Hong Kong in 1997 and subsequently re-emerged in Vietnam in 2003. From this time onwards it has rapidly spread across the globe and is likely to be endemic in poultry in many parts of the world. Although onward transmission to humans at present remains limited, the high case fatality rate in those people that are infected has raised concerns about the impact of a potential human pandemic [1], [2]. Whilst much research and planning is currently underway to contain any outbreak in humans, relatively little is known about the extent of infection in poultry and, in particular, the transmissibility of highly pathogenic avian influenzas between poultry farms. Such understanding is vital if we are to limit the potential for a human pandemic by reducing the extent of infection in poultry, either through movement restrictions, culling or vaccination.

Avian influenza occurs naturally in wild water fowl, usually in a low-pathogenic version (LPAI) causing no symptoms or only mild disease. However, in poultry some strains also occur in a highly-pathogenic form (HPAI) and result in a devastating disease which can kill up to 100% of infected birds within 48 hours, and is highly transmissible between individual birds [3], [4]. Transmission between flocks kept at different farms is thought to occur via movement of infected birds, equipment or staff, with current evidence suggesting that air-borne transmission over long distances is rare [5]. There has been an increase in HPAI outbreaks over the past ten years [4]. In addition to their implications for human health, these outbreaks also have severe economic consequences for the affected countries. Typical control measures for HPAI in poultry comprise of swift isolation and culling of flocks on infected farms, the restriction of movements between farms, increased bio-security, and the culling of flocks in the vicinity of infected farms to deplete the susceptible poultry population. Vaccination, if coupled with a strict surveillance programme, has also been demonstrated to be effective in reducing the risk of further outbreaks [5], [6].

The reproductive number for infected poultry farms, defined as the average number of farms that each original infected farm infects at the start of an outbreak (i.e., when most farms are susceptible), is an important measure of the overall transmissibility of the virus in a population. It determines whether a self-sustaining epidemic will occur and, more importantly, yields a tool to assess the effectiveness of control measures. If, on average, at any point in time, each infected farm infects more than one further farm, the epidemic will continue. However, if on average, each infected farm infects less than one further farm, the epidemic will decline and the intervention measures applied at that point can be interpreted as being sufficient to control the outbreak.

In this paper, we analyse published data from four outbreaks of HPAI in commercial poultry in industrialised countries to estimate the farm-to-farm reproductive number of HPAI to explore the extent to which different intervention measures implemented during these outbreaks reduce the reproductive number. The results from our analyses can be used to inform current planning for an outbreak of HPAI in similar commercial poultry sectors.

## Methods

### 2.1. Highly Pathogenic Avian Influenza outbreaks

**Figure 1:**
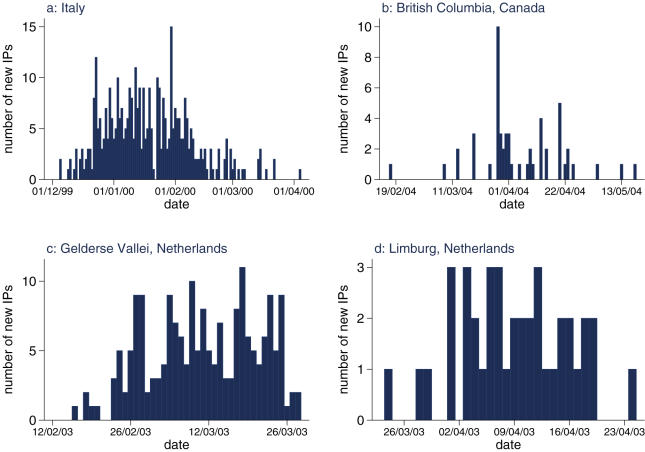
Time course of the four distinct HPAI epidemics considered. Number of infected farms detected daily for the four distinct epidemics. The data is given in Table S1.

We analyse data from three different outbreaks of HPAI that occurred in the past 8 years in industrialised countries: an outbreak of H7N1 in Italy in 1999/2000, an outbreak of H7N7 in the Netherlands in 2003, that will be treated as two distinct outbreaks due to geographic separation, and an outbreak of H7N3 in Canada in 2004. Figure 1 shows the time course of these outbreaks. Brief details of these outbreaks are given below.

#### 2.1.1. Outbreak of H7N1 in Italy in 1999/2000

Northern Italy has experienced a number of avian influenza outbreaks from 1997 onwards [6]–[10]. These all occurred in an extremely dense poultry production area (up to 70 000 birds/km^2^) and involved a significant number of farms keeping turkeys, a species known from experimental studies to be highly susceptible to avian influenza [11]. Furthermore, in this region there are many wetlands and resting sites for migratory waterfowl in close proximity to the poultry industry, which likely lead to multiple introductions from the wild bird host.

In March 1999, H7N1 LPAI was detected in a farm keeping turkeys [6], [7], [9]. This outbreak was not controlled rigorously and so AI continued to circulate. In December, a case of H7N1 HPAI was found and strict control measures were implemented, including culling of affected flocks, movement restrictions and pre-emptive slaughter of flocks deemed at high risk. However, due to LPAI circulating at the time, the confirmation of HPAI was delayed, and so the disease had already infected a number of farms by the time control measures were enforced. This resulted in an HPAI epidemic affecting a total of 413 flocks. The LPAI/HPAI epidemic lasted until April 2000, and involved a total of over 13 million birds.

#### 2.1.2. Outbreak of H7N7 in the Netherlands in 2003

The H7N7 epidemic in the Netherlands in 2003 affected a total of 255 commercial flocks in two distinct geographical and temporal clusters. The outbreak was situated in the Gelderse Vallei, the densest poultry production area in the Netherlands, in which over 10 million birds are kept in 984 flocks with a density of 4 flocks/km^2^ [12], [13]. Two months into the outbreak the infection passed to Limburg, another very dense poultry production area, where it continued to spread.

In the Gelderse Vallei, HPAI was confirmed on 28^th^ February, 6 days after clinical signs appeared in the first infected farm, and between March and early April, a total of 212 farms were infected. In Limburg, a further 43 farms were infected between April and early May.

A number of control policies were enforced in several stages. From 1^st^ March all movement of poultry and poultry products was banned, the tracing of dangerous contacts was initiated and reinforcement of strict bio-security measures was implemented. Two days later, from 3^rd^ March, culling of infected farms was initiated. On 5^th^ March the additional pre-emptive culling of farms within a 1 km radius of any infected farms was put in place. This was further extended to a 10 km radius for turkey flocks and 3 km radius for all other flocks on 7^th^ April [14]. However, these control measures were insufficient and it is hypothesized that the epidemics in both areas finally came to a halt due to depletion of susceptible flocks, after the culling of 30 million birds in 1,255 commercial and 17,421 hobby flocks [15].

During this epidemic, 89 human infections were also reported, most of whom presented with conjunctivitis or mild influenza-like illness. One person died from their infection. There was also evidence of limited human-to-human transmission [16], [17].

#### 2.1.3. Outbreak of HPAI H7N3 in British Columbia, Canada in 2004

The H7N3 outbreak in the Fraser Valley, British Columbia in Canada [18] started in February 2004 and lasted until mid-May. During the course of this outbreak, 42 commercial farms and 11 backyard flocks became infected, and a total of around 17 million commercial poultry were slaughtered which represented approximately 90% of the poultry population in the area.

Following detection of the index case, a broiler breeder farm, a surveillance program was initiated, which led to the detection of the second case on 11^th^ March. The Fraser Valley south of the River Fraser was declared a Control Area, restricting movements of birds, bird products and equipment. Furthermore, active surveillance was undertaken in a High Risk Region (HRR, 5 km around the index case) and in flocks deemed dangerous contacts in a Surveillance Region (SR, 10km around index case).

After the identification of 7 infected farms, all birds within the HRR were slaughtered from 24^th^ March onwards, but as this failed to stop transmission, on 5^th^ April it was decided to depopulate the whole Control Area, containing approximately 19 million birds.

Infected farms were located mainly in three distinct local clusters within the Control Area. It is hypothesized that long distance spread between these clusters was due to bird, equipment or people movement, whereas once a farm in a densely populated area became infected, where sheds are sometimes within a few hundred metres from each other, the virus spread via dust or feather debris.

### 2.2 Statistical Estimation of the Reproductive Number

Assuming homogeneous mixing, that all farms are equally infectious, and that the time-dependence of infectiousness from the point of infection is identical, we can estimate both the distribution of generation time intervals and the reproductive numbers of individual farms from the time-course of an epidemic using the following method [19].

Suppose there are *N* infected farms, labelled *i* = 1,…,*N*, and ordered so that the first *k* farms are those that contracted their infection from outside sources. The infection times of these farms are *t* = (*t*_1_,…,*t_N_*) such that *t*_1_ = … = *t_k_* = 0. Under the simplest model that neglects any differences between farms, spatial locations, etc., the probability that farm 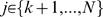 was infected by farm 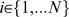 is
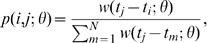
1where the generation time distribution has density *w*(*T;θ*), which is defined to be 0 if *T*<0 and indexed by unknown parameter vector *θ*. Under the above assumptions, the number of farms infected by farm *i* (i.e. the reproductive number of farm *i*) in the outbreak can be represented as an outcome from a random variable
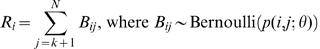
2that is, a sum of Bernoulli random variables, which has expected value 
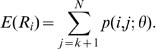
3


Now denote the ‘infection tree’ by *v* = (*v_k_*_+1_,…,*v_N_*), defined such that *v_j_* = *i* if farm *j* was infected by farm *i*. Under (1), the likelihood for *θ* when *v* and *t* are observed is
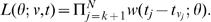
4


But as *v* is unobserved we sum over all possible infection trees to obtain the ‘integrated likelihood’
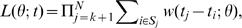
5where *S_j_* = {1,…,*N*}\{*j*} is the set of all indices other than *j*. The integrated likelihood is a genuine likelihood (up to a multiplicative constant) permitting valid inferences about *θ* conditional on outbreak size *N*.

#### 2.2.1. Maximum Likelihood estimation of the generation time

The maximum likelihood (ML) estimate 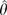 is obtained by minimizing twice the negative log-likelihood
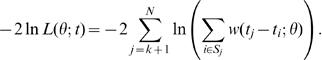
6


More details on how ML estimation is performed are given in Appendix S1. We assume the generation times *T* = *t_j_*−*t_i_* are Weibull distributed, with density
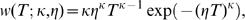
7and so *θ* = (*κ*, *η*). Minimization was performed using the Downhill Simplex method [20], the code used for these calculations is given in Code S1 and Code S2; to ensure the global minimum is reached, the procedure was run from 10000 different starting points.

We further investigated whether the generation time distribution changed after control measures were introduced. To do this, we extended the above model to allow for distinct parameters for the generation times before and after controls, *θ_pre_* and *θ_post_*, see Code S3 and Code S4. The improvement in fit compared to the original model was assessed using a likelihood ratio test.

#### 2.2.2. Estimation of the reproductive number

Given 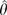 we can estimate the mean and variance of the generation time distribution. Moreover, we can estimate the reproductive number for each infected farm via equation (3), and the mean reproduction number for any subset of infected farms.

To calculate confidence intervals for the reproductive number we use an approximation of the parametric bootstrap percentile interval method [21]. To obtain proper parametric bootstrap intervals would involve generating infection times and trees according to the underlying epidemic model, which we do not wish to specify completely. Instead, the following two-step approximation is used, which we propose will be a good approximation for large *N* . These two steps approximate generating realisations from the underlying epidemic process. First, we take bootstrap samples of parameter values from the conventional approximation to the sampling distribution of the ML estimator,
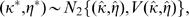
8that is, from a bivariate normal distribution with mean 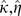 and variance-covariance matrix *V* based on the inverse of the observed information matrix (see Appendix S1 for further details, the code used to generate the bootstrap sample is given in Code S5 and Code S6).

This first stage can be loosely thought of as sampling the mean behaviour for a subgroup of possible outbreaks. To allow for variability within each subgroup, stage two involves fixing (*κ* ^*^,*η* ^*^)and independently generating reproductive numbers for each farm according to model (2). Steps one and two together give R^*^ = {R^*^_i_∶*i*−1,…,*N*}, an approximate bootstrap sample of the reproductive numbers for each farm. Here, 1000 samples of (*κ,η*)-pairs were drawn, and 500 sets of reproductive numbers generated for each. Finally, the approximate 95% CI for each *R_i_* is given by the 2.5^th^ and 97.5^th^ percentiles of the bootstrap distribution. The second stage of the calculation of the approximate CIs was done using Code S7 and Code S8.

## Results

### 3.1. Generation time distribution

The generation time is defined as the time between the infection of a farm and the time at which the farm passes on infection to another farm. We have assumed the generation time distribution is Weibull. While this is a biologically plausible choice, we cannot verify it empirically. As such, we assessed robustness to this choice using other plausible choices such as the gamma distribution (results not shown). However, the following results under these alternatives did not differ substantively from those shown below.

**Figure 2:**
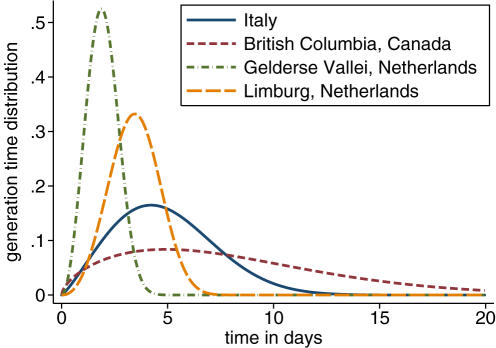
Maximum likelihood estimates for the generation time distributions. Parameters of these distributions are shown in Table 1.

Figure 2 shows the estimated generation time distribution for the four different outbreaks; the parameter estimates are detailed in Table 1. The estimates, and hence the distribution, differs substantially between the outbreaks. It could be hypothesised that the generation time would shorten after measures were put in place to isolate the infected farms. However, allowing for different generation time distributions for the pre- and post-control time periods did not significantly improve the model fit.

**Table 1:** Parameters of the generation time distributions.

Dataset	*κ*	*η*	mean	variance
Italy	2.2 (1.7–3.0)	0.18 (0.14–0.22)	5.0 (4.0–6.3)	5.9 (3.5–11.7)
British Columbia	1.6 (1.1–2.5)	0.11 (0.07–0.16)	8.4 (5.7–12.1)	29 (12–84)
Gelderse Vallei	2.9 (1.9–4.1)	0.46 (0.29–0.59)	1.9 (1.5–3.1)	0.51 (0.23–2.2)
Limburg	3.3 (1.6–8.3)	0.26 (0.19–0.38)	3.4 (2.3–4.9)	1.3 (0.37–5.1)

Shown are the maximum likelihood estimates (95% confidence intervals) of the parameters *κ* and *η*, and of mean and variance of the resulting distributions.

### 3.2. Estimates of the reproductive number

**Figure 3:**
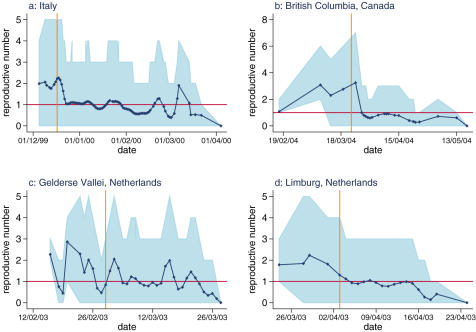
Estimates of the reproductive number over time for the four epidemics. The estimates are obtained with the MLE for the generation time distribution parameters. The light blue area shows the 95% confidence intervals. The vertical orange line marks the date of reinforced controls. For British Columbia, this was the date the decision to cull the HRR region was taken, for the other datasets it is the date of detection of HPAI within the area.

Figure 3 shows the maximum likelihood estimates and their 95% confidence intervals of the farm-based reproductive numbers over the course of the outbreaks. For all four outbreaks the estimates of the mean reproductive number prior to the controls being implemented are between 1.1 and 2.4 (Table 2) with upper 95% bounds in the range 1.5–3.6.

**Table 2:** Maximum and mean reproductive numbers for each outbreak.

Dataset	Maximum *R*	mean *R* pre-intervention
Italy	2.3	1.9 (1.2–2.7)
British Columbia	3.2	2.4 (1.4–3.6)
Gelderse Vallei	2.9	1.1 (0.9–1.5)
Limburg	2.2	1.9 (1.0–3.0)

Maximum reproductive numbers estimated for any farm during the course of each outbreak and mean reproductive numbers *R* (95% confidence intervals) prior to enforced interventions for the four different datasets. The mean reproductive numbers prior to intervention were calculated using Code S9 and Code S10.

The impact of control measures on the effective reproductive number can be clearly seen in all four outbreaks. For the outbreak in Italy (Figure 3a), their introduction rapidly reduced the reproductive number, hovering around the threshold of 1 for the next few months before finally dying out. In British Columbia (Figure 3b) controls were put in place after detection of the first IP. However our estimates of the reproductive number remain high until 24^th^ March when the decision was taken to cull the whole high risk region. Our estimates show that the control activities following this decision were effective in reducing the reproductive number to below one.

Our results show that the situation in the Gelderse Vallei, The Netherlands (Figure 3c) differed in that the initial control measures failed to bring the reproductive number reliably below 1, and the epidemic only died out at the end of March after the depletion of susceptible flocks in the affected area [15]. The same controls were applied to the Limburg epidemic but our estimates show in this case the reproductive number was reduced to just below 1 (Figure 3d), and so potentially effective in controlling the outbreak. However, the end of the epidemic in late April coincided here too with the depletion of susceptible flocks and therefore it is possible that the epidemic would have taken substantially longer to control had there been a larger pool of susceptible flocks in the area.

## Discussion

Our estimates of the farm-to-farm reproductive number prior to interventions for HPAI are in the range 1.1 to 2.4 and were remarkably consistent across the four datasets. However, these estimates are substantially lower than those previously reported for the Dutch epidemic. Prior to the implementation of control measures we obtained estimates of 1.1 (95% CI 0.9–1.5) in the Gelderse Vallei and 1.9, (95% CI 1.0–3.0) in Limburg which are significantly lower than those previously reported for the same outbreak prior to notification (6.5 (95% CI 3.1–9.9) for the epidemic in the Gelderse Vallei). However, as demonstrated in Figure 3c, there was substantial variation in our estimates of individual reproductive numbers prior to interventions. In addition, in the previous study, the generation time was not estimated directly from the data but based on observational and experimental data on the course of infection in the farms. Our estimate of the generation time for this region is of the order of 2 days, whereas the values previously assumed for the infectious period were defined per flock as the time between detection and culling plus an additional 4 days to cover the time before the infection was detected but during which birds were infectious. The previously published estimates therefore assumed a much longer mean generation time and this could also lead to a higher estimated reproductive number.

Our results showed substantial differences between the estimated generation time distributions for the different outbreaks. Whilst much is known from experimental studies on the course of infection in individual birds [11], [22]–[24], estimates of the generation time at the farm-level are more difficult to obtain. Although it is perhaps surprising that the generation time differs between the outbreaks, it is plausible that such differences could arise because of variation in farming practices or in the contact patterns between farms. In addition, the latent and infectious periods determining the generation time may differ by the strain of HPAI. Alternatively, the estimates may be biased because of assumptions made in the method. In particular, we assumed that the datasets were complete (and thus that all infected farms were detected) and that only the first farm in each outbreak was infected from an outside source. If, however, further undetected farms had played a role in transmission, this would substantially alter the estimates of the generation time and the reproductive number, particularly if these infections occurred towards the beginning or end of the epidemics where overall cases are sparser.

All of the outbreaks investigated here occurred within dense poultry farming areas and hence were difficult to control. The control policies implemented in the different outbreaks were similar, comprising strict bio-security measures for movement of poultry and poultry products, swift culling of infected flocks, and if these failed to control the epidemics, additional pre-emptive culling of flocks in the neighbourhood of any infected farms. Our results demonstrate that the bio-security measures, movement restrictions and culling of infected farms, all of which were initiated early on in the outbreaks, did have an effect but for all four outbreaks only reduced the reproductive number to close to the threshold value of 1. The additional pre-emptive culling of flocks and de-population of the areas was needed to fully control the outbreaks. Current contingency plans for HPAI outbreaks in Europe focus on the former set of control measures to contain any outbreak [25]. Whilst differences in farming practices between countries mean that it is difficult to predict whether these measures will be sufficient for a new outbreak, our analyses suggest that additional interventions may well be required. Close monitoring of outbreaks, coupled with quantitative estimation of the reproductive number, is therefore needed to ensure that such additional measures, if required, are promptly implemented.

The method used here to estimate the reproductive number and generation time parameters is an extension of that developed by Wallinga and Teunis [19] for the SARS-epidemic. This method requires only time-series data for an outbreak, and is therefore easily applied even in real-time. Technically, appropriate censoring terms should be added to the likelihood to account for infection times yet to occur, but a straightforward application of the method as described here will give estimates unbiased in an asymptotic sense. If data on the spatial location of infected farms are also available, this information can easily be incorporated to estimate the spatial transmission kernel and improve the estimation of the reproductive numbers. Such an approach was successfully applied to the Foot-and-Mouth epidemic in the UK in 2001 [26]. Further work is required to explicitly incorporate missing data, as this is likely to have a strong influence on the estimates of both the generation time and the reproductive number. Such methods are of particular importance to estimating the reproductive number for outbreaks of HPAI in Asia in which, with high general levels of poultry mortality, cases are likely to be less well documented.

## Supporting Information

Appendix S1 The Transmissiblity of Highly Pathogenic Avian Influenza in Commercial Poultry in Industrialised Countries: Technical Appendix
(0.07 MB PDF)

Table S1 Time Series of the Epidemics
(0.00 MB TXT)

Code S1 Source code for the maximum likelihood estimation of the parameters of the generation time distribution, shown in Figure 2 and Table 1.
(0.02 MB TXT)

Code S2 header file for Code S1
(0.00 MB TXT)

Code S3 Source code for the maximum likelihood estimation of the parameters of the generation time distribution, assuming two different distributions (i.e., different parameters), pre- and post-intervention.
(0.02 MB TXT)

Code S4 header file for Code S3
(0.00 MB TXT)

Code S5 Source code for generating the bootstrap samples for the parameters of the generation time distribution, used in the calculation of the confidence intervals.
(0.01 MB TXT)

Code S6 header file for Code S5
(0.00 MB TXT)

Code S7 Source code for estimating R0 and the confidence intervals based on the uncertainties inherent in the estimation procedure and the uncertainty of the generation time distribution, shown in Figure 3.
(0.02 MB TXT)

Code S8 header file for Code S7
(0.00 MB TXT)

Code S9 Source code for calculating the mean R0 prior to interventions and its confidence intervals shown in Table 2.
(0.01 MB TXT)

Code S10 header file for Code S9
(0.00 MB TXT)
