# Supplementary material for: The Transmissibility of Highly Pathogenic Avian Influenza in Commercial Poultry in Industrialised Countries
Source: PLoS One. 2007 Apr 4;2(4):e349. doi: 10.1371/journal.pone.0000349 (PMC1831494; doi:10.1371/journal.pone.0000349)
Supplement: Appendix S1 — The Transmissiblity of Highly Pathogenic Avian Influenza in Commercial Poultry in Industrialised Countries: Technical Appendix (0.07 MB PDF) [file pone.0000349.s001.pdf]

The Transmissibility of Highly Pathogenic Avian Influenza in Commercial Poultry in Industrialized Countries

Tini Garske, Paul Clarke, Azra C. Ghani

Technical Appendix

1 The information matrix for discrete times

In the main paper, we assumed that distribution times followed a Weibull distribution which is a continuous distribution. However, our infection time data is discretized into whole days, and the likelihood must be discretized to reflect this.

To calculate the maximum likelihood (ML) estimate, substitute $W(t_j - t_i; \kappa, \eta)$ for $w(t_j - t_i; \kappa, \eta)$ in equation (6) of the main paper. The Weibull cumulative distribution function (CDF) is given by

$$W(T; \kappa, \eta) = 1 - \exp\left[-(\eta T)^\kappa\right] , \tag{A.1}$$

and for every occurrence of $w(t_j - t_i; \kappa, \eta)$ we substituted

$$\begin{aligned} w_{ij} &= W(T + 1/2; \kappa, \eta) - W(T - 1/2; \kappa, \eta) && \text{(A.2)} \\ &= \exp\left[-\left(\eta(T - 1/2)\right)^\kappa\right] - \exp\left[-\left(\eta(T + 1/2)\right)^\kappa\right] && \text{(A.3)} \\ &= E^- - E^+ . && \text{(A.4)} \end{aligned}$$

To calculate variance-covariance matrix in equation (8) of the main paper, we use the standard relationship

$$V(\theta) = J^{-1}(\theta) \tag{A.5}$$

where $J(\theta)$ is the observed information matrix given by

$$J(\hat{\theta}) = -\begin{pmatrix} \frac{\partial^2 \ln L}{\partial \kappa^2} & \frac{\partial^2 \ln L}{\partial \kappa \partial \eta} \\ \frac{\partial^2 \ln L}{\partial \eta \partial \kappa} & \frac{\partial^2 \ln L}{\partial \eta^2} \end{pmatrix} \tag{A.6}$$

with $\hat{\theta} = (\kappa, \eta)$ and $\ln L$ the log-likelihood with the cumulative density for the Weibull substituted.

To calculate J it is first necessary to evaluate the first and second derivatives with respect to κ and η, given by

$$\frac{\partial}{\partial\kappa}E^{\pm} = \beta_{\kappa\pm}E^{\pm} \tag{A.7}$$

$$\frac{\partial}{\partial\eta}E^{\pm} = \beta_{\eta\pm}E^{\pm} \tag{A.8}$$

$$\frac{\partial^2}{\partial\kappa^2}E^{\pm} = \beta_{\kappa\kappa\pm}E^{\pm} = \left(\beta_{\kappa\pm}^2 + \frac{\partial}{\partial\kappa}\beta_{\kappa\pm}\right)E^{\pm} \tag{A.9}$$

$$\frac{\partial^2}{\partial\eta\partial\kappa}E^{\pm} = \beta_{\kappa\eta\pm}E^{\pm} = \left(\beta_{\kappa\pm}\beta_{\eta\pm} + \frac{\partial}{\partial\eta}\beta_{\kappa\pm}\right)E^{\pm} \tag{A.10}$$

$$\frac{\partial^2}{\partial\eta^2}E^{\pm} = \beta_{\eta\eta\pm}E^{\pm} = \left(\beta_{\eta\pm}^2 + \frac{\partial}{\partial\eta}\beta_{\eta\pm}\right)E^{\pm}\,. \tag{A.11}$$

With $T^{\pm} = T \pm 1/2$, we have

$$\beta_{\kappa\pm} = -\left(\eta T^{\pm}\right)^{\kappa}\ln\left(\eta T^{\pm}\right) \tag{A.12}$$

$$\beta_{\eta\pm} = -\frac{\kappa}{\eta}\left(\eta T^{\pm}\right)^{\kappa} \tag{A.13}$$

$$\beta_{\kappa\kappa\pm} = \beta_{\kappa\pm}^2 - \left(\eta T^{\pm}\right)^{\kappa}\ln^2\left(\eta T^{\pm}\right) \tag{A.14}$$

$$\beta_{\kappa\eta\pm} = \beta_{\kappa\pm}\beta_{\eta\pm} - \frac{\left(\eta T^{\pm}\right)^{\kappa}}{\eta}\left(1 + \kappa\ln\left(\eta T^{\pm}\right)\right) \tag{A.15}$$

$$\beta_{\eta\eta\pm} = \beta_{\eta\pm}^2 - \frac{\kappa}{\eta^2}\left(\eta T^{\pm}\right)^{\kappa}(\kappa - 1)\,. \tag{A.16}$$

Therefore the entries of the information matrix are

$$\frac{\partial^2 \ln L}{\partial \kappa^2} = \sum_{j=k}^{N} \left[\frac{\sum_{i \in S_j} (\beta_{\kappa\kappa -} E^- - \beta_{\kappa\kappa +} E^+)}{\sum_{i \in S_j} (E^- - E^+)} - \frac{\left(\sum_{i \in S_j} (\beta_{\kappa -} E^- - \beta_{\kappa +} E^+)\right)^2}{\left(\sum_{i \in S_j} (E^- - E^+)\right)^2} \right] \tag{A.17}$$

$$\frac{\partial^2 \ln L}{\partial \kappa \eta} = \sum_{j=k}^{N} \left[\frac{\sum_{i \in S_j} (\beta_{\kappa\eta -} E^- - \beta_{\kappa\eta +} E^+)}{\sum_{i \in S_j} (E^- - E^+)} - \frac{\left(\sum_{i \in S_j} (\beta_{\kappa -} E^- - \beta_{\kappa +} E^+)\right)\left(\sum_{i \in S_j} (\beta_{\eta -} E^- - \beta_{\eta +} E^+)\right)}{\left(\sum_{i \in S_j} (E^- - E^+)\right)^2} \right] \tag{A.18}$$

$$\frac{\partial^2 \ln L}{\partial \eta^2} = \sum_{j=k}^{N} \left[\frac{\sum_{i \in S_j} (\beta_{\eta\eta -} E^- - \beta_{\eta\eta +} E^+)}{\sum_{i \in S_j} (E^- - E^+)} - \frac{\left(\sum_{i \in S_j} (\beta_{\eta -} E^- - \beta_{\eta +} E^+)\right)^2}{\left(\sum_{i \in S_j} (E^- - E^+)\right)^2} \right] \tag{A.19}$$

Once this is done standard formulae for a) the inverse of a two-by-two matrix and b) the conditional normal distribution, can be used to calculate $V = J^{-1}$ and generate draws from the bivariate normal distribution. For completeness, we give each of these results below.

2 Inversion of the information matrix

The variance-covariance matrix is obtained by inverting the information matrix. The general inversion formula for a 2×2 matrix

$$M = \begin{pmatrix} A & B \\ C & D \end{pmatrix} \tag{A.20}$$

is given by

$$M^{-1} = \begin{pmatrix} \frac{D}{\det} & -\frac{B}{\det} \\ -\frac{C}{\det} & \frac{A}{\det} \end{pmatrix} \quad \text{with} \quad \det = AD - BC\,. \tag{A.21}$$

3 Marginal and conditional distributions

The distribution function of a bivariate normal distribution is given by

$$P(x_1, x_2) = \frac{1}{2\pi\sigma_1\sigma_2\sqrt{1-\rho^2}} \exp\left[-\frac{z}{2(1-\rho^2)}\right] \tag{A.22}$$

with

$$z = \frac{(x_1-\mu_1)^2}{\sigma_1^2} - \frac{2\rho(x_1-\mu_1)(x_2-\mu_2)}{\sigma_1\sigma_2} + \frac{(x_2-\mu_2)^2}{\sigma_2^2}\,. \tag{A.23}$$

The variance-covariance matrix V determines the variances and correlations via

$$V = \begin{pmatrix} \Sigma_{11} & \Sigma_{12} \\ \Sigma_{21} & \Sigma_{22} \end{pmatrix} = \begin{pmatrix} \sigma_1^2 & \rho\sigma_1\sigma_2 \\ \rho\sigma_1\sigma_2 & \sigma_2^2 \end{pmatrix}\,. \tag{A.24}$$

The marginal probability for any x_i is given by the univariate normal distribution

$$P(x_i) = \frac{1}{\sigma_i\sqrt{2\pi}} \exp\left[-\frac{(x_i-\mu_i)^2}{2\sigma_i^2}\right]\,. \tag{A.25}$$

The conditional probability of x_2 given that $x_1 = a$ is also a normal distribution, but with mean $\bar{\mu_2}$ and variance $\bar{\sigma_2^2}$, which are given as

$$\bar{\mu}_2 = \mu_2 + \frac{\Sigma_{21}}{\Sigma_{11}}(a-\mu_1) \tag{A.26}$$

$$\bar{\sigma}_2^2 = \Sigma_{22} - \frac{\Sigma_{21}\Sigma_{12}}{\Sigma_{11}} \tag{A.27}$$
